# Correlation Studies Between Double-Stranded DNA and Diabetes Mellitus

**DOI:** 10.1155/jdr/9919456

**Published:** 2025-04-29

**Authors:** Wanli Niu, Zhuo Li, Chunmeng Liu, Ziyan Zhang, Weiwei Chen, Yirui Wang, Xiaofen Guo, Xinyu Feng, Yuge Wang, Guanglei Shi, Yuhang Liu, Haoran Shen, Yang Han, Qi Zhen, Ruimin Wang, Liangdan Sun

**Affiliations:** ^1^School of Basic Medicine, North China University of Science and Technology, Tangshan, Hebei, China; ^2^Hebei Key Laboratory for Chronic Diseases, School of Basic Medicine, North China University of Science and Technology, Tangshan, China; ^3^Department of Dermatology, the First Affiliated Hospital of Anhui Medical University, Hefei, China; ^4^School of Public Health, North China University of Science and Technology, Tangshan, Hebei, China; ^5^North China University of Science and Technology Affiliated Hospital, Tangshan, China; ^6^School of Clinical Medicine, North China University of Science and Technology, Tangshan, Hebei, China; ^7^Key Laboratory for quality of salt alkali resistant TCM of Hebei Administration of TCM, North China University of Science and Technology, Tangshan, Hebei, China

## Abstract

**Background:** Diabetes mellitus (DM) is a common chronic endocrine and metabolic disease, and its complications can involve multiple organs and seriously threaten human health. Double-stranded DNA (dsDNA) plays an important role in the autoimmune system; however, the correlation between dsDNA and DM has not been fully studied.

**Methods:** This study recruited 388 diabetic patients and 2970 healthy controls to investigate the relationship between serum dsDNA and DM. The diagnosis of DM was based on the medical diagnostic and treatment standards for DM published by the American Diabetes Association (ADA). The study adhered to ethical principles and obtained informed consent from all participants. We measured serum dsDNA levels in both diabetic patients and healthy controls. The study examined differences in serum dsDNA levels among diabetic patients under various conditions, including different temperatures, ultraviolet (UV) light exposure, seasons, and clinical indicators. Additionally, quantitative PCR was used to assess the expression of dsDNA receptors, single-stranded RNA (ssRNA) receptors, absent in melanoma factor 2 (AIM2)–related inflammatory factors, and Type I interferon (INF) in the peripheral blood of patients and control groups.

**Results:** Peripheral blood serum dsDNA levels were elevated in diabetic patients compared to controls (mean values 1.09 and 0.97 ng/ml, respectively, *p* < 0.001). We also found that the gene expression levels of dsDNA receptor, ssRNA receptor, AIM2-related inflammatory factors, and Type I IFN in diabetic patients were upregulated. And serum dsDNA levels correlated with clinical indicators.

**Conclusions:** We have confirmed that DM is closely associated with serum dsDNA levels. Therefore, dsDNA detection shows promise as a novel approach for evaluating DM progression, offering new insights for the future diagnosis and treatment of DM.

## 1. Introduction

Diabetes mellitus (DM) is a metabolic disease that can lead to pathological changes in multiple organs. Its pathogenesis is complex and includes genetic factors, environmental factors, and defects in the autoimmune system [1, 2]. Ultraviolet (UV) radiation has been demonstrated to directly damage DNA, which may subsequently trigger autoimmune responses [3]. Temperature changes affect the stability and structure of double-stranded DNA (dsDNA), and these alterations may influence the immunogenicity of DNA [4]. Furthermore, seasonal variations may indirectly modulate dsDNA levels by impacting immune system function [5]. These environmental factors could significantly affect the biological functions of dsDNA and its role in autoimmune diseases. It is estimated that 670,000 people worldwide die from DM and its complications, making DM an increasingly significant global public health issue [6].

Circulating free DNA (cfDNA) is DNA that exists freely in the peripheral blood, including single-stranded DNA (ssDNA) and dsDNA. The 5-hydroxymethylcytosine (5hmC) profile of cfDNA can provide early predictive markers for DM diagnosis and serves as a biomarker for autoimmune disease detection [7–9]. Hyperglycemia associated with DM can alter neutrophil function, leading to abnormal neutrophil activation and the formation of neutrophil extracellular traps (NETs). NETs consist of a dsDNA backbone embedded with granule proteins and histones in a web-like structure. Excessive aggregation of NETs or impaired clearance mechanisms may harm the body, potentially leading to inflammatory diseases [10–13]. Therefore, the relationship between DM and dsDNA needs to be further studied.

DM has become a significant global issue. To reduce the occurrence of diabetic complications, this study investigates the differences in serum dsDNA levels between diabetic patients and healthy volunteers, further exploring the role and mechanism of dsDNA in DM.

## 2. Materials and Methods

### 2.1. Source and Handling of Samples

This study recruited 388 diabetic patients admitted to a hospital in Eastern China. The diagnosis of DM was based on the medical diagnostic and treatment standards for DM published by the American Diabetes Association (ADA), and all patients were mentally normal. Additionally, 2970 healthy Chinese volunteers were recruited, who were in good health and free from DM and other autoimmune diseases. This study was approved by the hospital ethics committee, and all participants provided written informed consent. Medical staff collected peripheral blood from patients and control subjects, followed by the separation of serum and plasma.

### 2.2. Initial Detection of Serum dsDNA

A fluorescence quantification method based on fluorescence detection combined with dye was used to measure serum dsDNA in peripheral blood. The dsDNA was detected and analyzed using an EzQuant quantitative analysis reagent (BioVision, catalog number: K900-2000-1, United States), and the experimental procedure is completed in accordance with the product instructions. The fluorescence value of each sample well at 28°C, EX/EM = 480/530 nm, was detected by Cell Imaging Multi-Mode Reader (BioTek, Cytation 5, United States), and the fluorescence value and standard curve of each well were calculated to obtain the concentration of dsDNA.

### 2.3. Relationship Between dsDNA and Meteorological Data

A retrospective analysis was conducted on patients attending the DM outpatient clinic at a hospital in East China to determine the onset time of the disease. By consulting the weather website (http://www.weather-atlas.com), the season, temperature, and UV index on the date of onset for each patient were recorded, and these climatic factors were categorized into season groups, temperature groups, and UV groups. Based on the meteorological data of the region, patients whose onset occurred in months with an average monthly temperature of 2.1°C–15.5°C (November to April) were defined as the low-temperature group. Patients whose onset occurred in months with an average monthly temperature of 17°C–28.3°C (May to October) were defined as the high-temperature group. Additionally, the daily UV index on the date of blood draw was obtained from the meteorological data and divided into the high-UV and low-UV groups. A UV index greater than or equal to 7 was classified as the high-UV group, while a UV index less than 7 was classified as the low-UV group.

### 2.4. RNA Isolation and Quantitative PCR

Using TRIzol reagent, follow the instructions to isolate total RNA from whole blood samples. RNA was reverse-transcribed to cDNA using the Evo M-MLV RT Kit with gDNA Clean for qPCR (Accurate Biology, Changsha, China). Quantitative PCR was performed using the SYBR Green Premix Pro Taq HS qPCR Kit (Accurate Biology) and the StepOnePlus Real-Time PCR System (Applied Biosystems). The relative expression of the gene was calculated using the 2^−ΔΔCT^ method and normalized by the housekeeping gene GAPDH. The baseline characteristics of gene expression measurements in diabetic patients and the control group are presented in Table 1.

### 2.5. Statistical Analysis

The MatchIt package in R language version 4.2.2 (https://www.r-project.org/) was used for propensity score matching (PSM) to adjust for potential bias. From the healthy control group, the gender and age matching control group was selected according to the ratio of 1:3. The analysis was based on a 95% confidence interval (CI), with a *p*-value < 0.05 defined as statistically significant. SPSS 23.0 software was used for statistical analysis of other data. The Mann–Whitney test compares the difference between two independent groups and applies to data that are not normally distributed.

## 3. Results

### 3.1. Serum dsDNA Is Elevated in Patients With DM

After eliminating cases with missing age or gender information from 388 diabetic patients, the total number of patients was 339.  Table 2 presents the data information of both diabetic patients and the normal control group, encompassing age, gender, season, temperature, and other characteristics. PSM was applied at a 1:3 ratio with a caliper of 0.2 to balance covariate differences between diabetic patients and healthy volunteers.  Table 2 shows the baseline characteristics of the pre- and postmatched cohorts, resulting in 339 diabetic patients matched to 904 controls.

### 3.2. Serum dsDNA Is Elevated in Diabetic Patients

**Figure 1:**
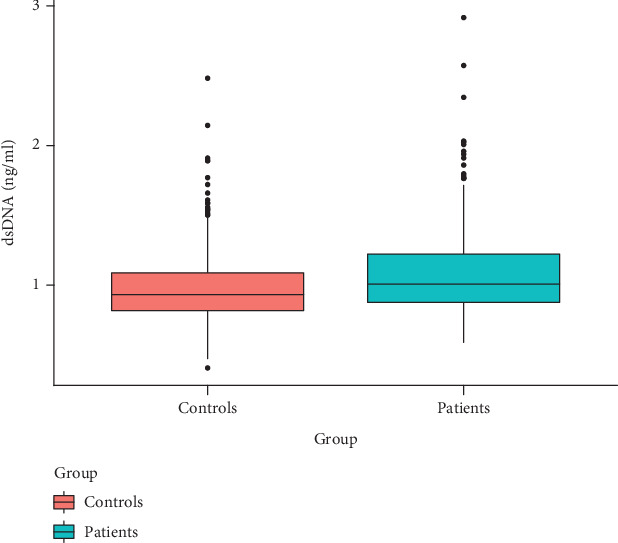
Box plot of serum dsDNA levels in diabetic patients and healthy controls.

Figure 1 illustrates the differences in serum dsDNA levels between diabetic patients and matched healthy controls. The results show that the median serum dsDNA level in 339 diabetic patients was 1.09 ng/ml, while the median serum dsDNA level in 904 matched healthy controls was 0.97 ng/ml. The median serum dsDNA level was significantly higher in diabetic patients compared to the control group, with a statistically significant difference (mean: 1.09 vs. 0.97, SD: 0.32 vs. 0.22, *p* < 0.001, Figure 1).

### 3.3. Increased Expression of dsDNA Receptor Genes in Diabetic Patients

**Figure 2:**
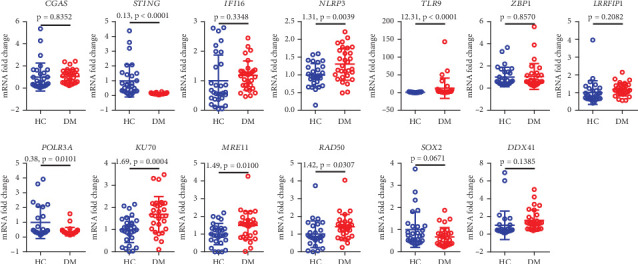
Serum dsDNA receptor gene expression in diabetic patients and healthy controls. HC, healthy control; DM, diabetes mellitus.

By employing real-time quantitative PCR to analyze serum dsDNA for receptor gene expression in 30 diabetic patients and 30 healthy controls, it was found that the expression of *STING*, *NLRP3*, *TLR9*, *POLR3A*, *KU70*, *MRE11*, and *RAD50* differed between the two groups. Specifically, the expression of TLR9 and *KU70* receptor genes was elevated in diabetic patients (*p* < 0.001), with TLR9 showing the most significant difference in gene expression between the two groups (Figure 2).

### 3.4. Increased Expression of ssRNA Receptor Genes in Diabetic Patients

**Figure 3:**
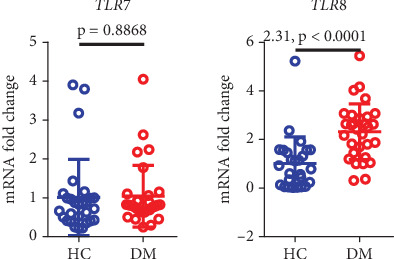
Expression of ssRNA receptor genes in diabetic patients and healthy controls. HC, healthy control; DM, diabetes mellitus.

Toll-like receptors (TLRs) are a class of immune recognition receptors whose activation triggers the release of inflammatory immune factors, which in turn participate in the immunomodulatory process. TLR7 and TLR8 exhibit a high degree of sequence homology and are capable of recognizing ssRNA [14]. We analyzed ssRNA receptor gene expression and found that the expression of the gene encoding TLR8 was higher in diabetic patients than in healthy controls (*p* < 0.001), but TLR7 was similarly expressed between the two groups (Figure 3).

### 3.5. Increased Expression of dsRNA Receptor Genes in Diabetic Patients

**Figure 4:**
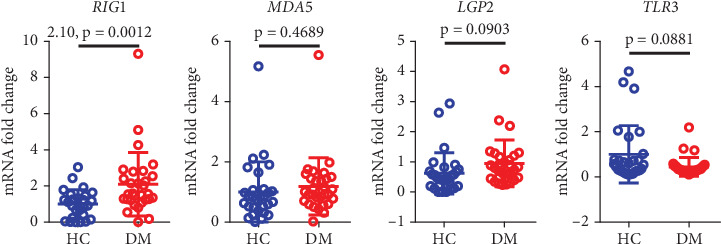
Expression of RIG1, MDA5, LGP2, and TLR3 in diabetic patients and the healthy control group. HC, healthy control; DM, diabetes mellitus.

Double-stranded RNA (dsRNA), upon binding to intracellular dsRNA receptors, activates multiple immunoregulatory pathways that play critical roles in viral defense and gene regulation [15]. We analyzed common dsRNA receptors and found a significant upregulation of the RIG1 receptor gene expression in diabetic patients (*p* < 0.01), but there was no significant correlation between MDA5, LGP2, and TLR3 between the two groups (Figure 4).

### 3.6. The Expression of Absent in Melanoma Factor 2 (AIM2)-Related Inflammatory Factors Was Increased in Diabetic Patients

**Figure 5:**
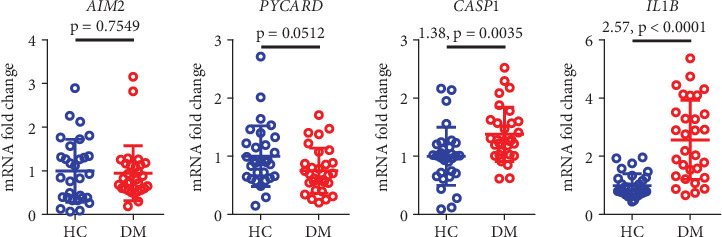
The expression differences of CASP1 and IL-1*β* between diabetic patients and healthy controls were statistically significant. Real-time PCR analysis was performed to examine the expression relationships of AIM2, PYCARD, CASP1, and IL-1*β* in the serum of diabetic patients and healthy controls. Data are presented as mean ± SD from three independent experiments. HC, healthy control; DM, diabetes mellitus.

By using real-time quantitative PCR to analyze the expression of AIM2-related inflammatory factors in the serum of 30 diabetic patients and 30 healthy controls, we found that the expression of AIM2 was similar between diabetic patients and healthy controls. However, the expression of Caspase-1 (CASP1) and IL-1*β* was increased in diabetic patients (Figure 5).

### 3.7. Type I Interferon Expression Is Elevated in Diabetic Patients

**Figure 6:**
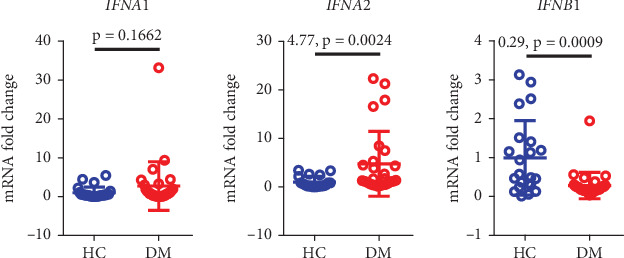
Differences in the expression of IFN-*α* and IFN-*β* between diabetic patients and healthy control groups. HC, healthy control; DM, diabetes mellitus.

Compared to healthy controls, diabetic patients showed significant differences in the expression levels of IFN-*α*2 (*p* = 0.0024) and IFN-*β*1 (*p* = 0.0009), but gene expression levels of IFN-*α*1 were not significantly different between the two groups (Figure 6).

### 3.8. Correlation Between Serum dsDNA and Different Clinical Indicators in Diabetic Patients

**Figure 7:**
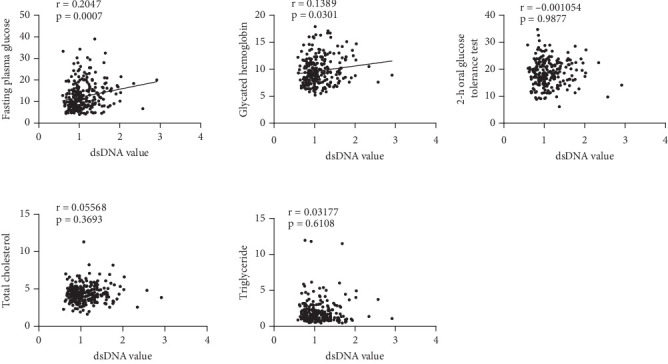
Comparison of serum dsDNA levels in diabetic patients across different clinical indicators.

We analyzed the differences in serum dsDNA levels among diabetic patients based on fasting plasma glucose (FPG), glycated hemoglobin (HbA1c), 2-h oral glucose tolerance test (2-h OGTT), total cholesterol (TC), and triglyceride (TG) indices, to determine whether there is a correlation between these clinical indicators and serum dsDNA levels. The study revealed that serum dsDNA levels were positively correlated with FPG (*r* = 0.2047, *p* = 0.0007) and HbA1c (*r* = 0.1389, *p* = 0.0301), with statistical significance (Figure 7).

### 3.9. Correlation of Serum dsDNA With Different Temperatures, UV Exposure, and Seasons in Diabetic Patients

**Figure 8:**
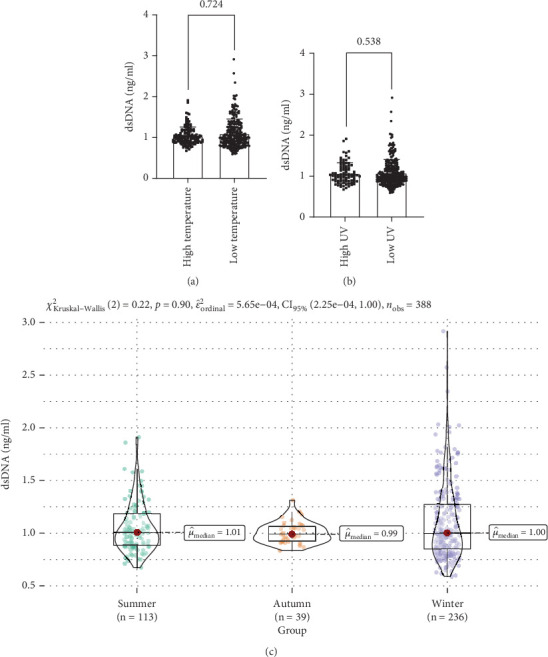
(a) Differences in serum dsDNA in diabetic patients at different temperatures; (b) differences in serum dsDNA in diabetic patients under different degrees of UV irradiation; (c) differences in serum dsDNA in diabetic patients in different seasons.

There was no significant relationship between serum dsDNA in diabetic patients across temperature, UV light, and season (Figure 8).

## 4. Discussion

DM is a complex, multiorgan disease whose pathogenesis has not yet been fully elucidated. In recent years, a growing body of research has indicated that the pathogenesis of DM may be closely associated with the immune system. When cells or mitochondria are damaged, dsDNA enters the cytoplasm or extracellular space, where it is recognized as damage-associated molecular patterns (DAMPs), thereby activating the immune system and participating in the host's immune regulation [16]. Currently, research on dsDNA primarily focuses on systemic lupus erythematosus (SLE), but there is relatively less research on its role in DM. We found a significant increase in dsDNA levels in peripheral blood serum of diabetic patients compared to the control group.

We observed that compared to the control group, diabetic patients exhibited upregulation in the expression levels of dsDNA receptors, ssRNA receptors, AIM2-related inflammatory factors, and Type I IFN genes. Cyclic GMP-AMP synthase (cGAS) is a cytosolic DNA sensor capable of recognizing aberrantly localized dsDNA. Upon binding to DNA, cGAS catalyzes the conversion of GTP and ATP into cyclic GMP-AMP (cGAMP). cGAMP binds to the stimulator of interferon genes (STING), inducing the expression of proinflammatory cytokines such as Type I IFN and interleukin-6 (IL-6), thereby participating in the inflammatory response [17–20]. Our study demonstrates that the expression level of STING in diabetic patients significantly differs from that in healthy controls. This finding is closely related to the critical role of the cGAS-STING signaling pathway in inflammatory responses. Alterations in STING expression levels may influence the regulation of inflammatory responses in diabetic patients, thereby contributing to the pathogenesis and progression of the disease.

TLRs are a type of pattern recognition receptors (PRRs) that can activate immune responses. This study focuses on the expression of TLR7, TLR8, and TLR9 in patients with DM. TLR9 is a DNA recognition receptor that can specifically recognize unmethylated cytosine-phosphone-guanine (CpG) DNA. TLR7 and TLR8 are capable of recognizing ssRNA, thereby initiating immune responses against pathogens [21–23]. Our results showed that there were significant differences in the expression levels of TLR9 and TLR8 in the peripheral blood of diabetic patients compared to healthy controls. The altered expression of TLR9 may affect the body's ability to recognize pathogen-derived DNA, while the aberrant expression of TLR8 could potentially disrupt antiviral immune responses. These changes may influence the recognition of pathogens and subsequent immune reactions, which could be associated with the pathogenesis and progression of DM and its complications.

AIM2 belongs to the PYHIN protein family and is highly expressed in various cell types, where it plays a role in inflammatory responses. Upon recognition of dsDNA, AIM2 activates CASP1, leading to the substantial release of downstream inflammatory cytokines IL-1*β* and IL-18, thereby inducing pyroptosis [24–26]. Our research has revealed that, compared to healthy controls, the expression levels of CASP1 and IL-1*β* are significantly elevated in diabetic patients, while the expression level of AIM2 shows no notable difference. This discovery suggests that there may be other inflammatory pathway activation mechanisms independent of AIM2 in the pathological process of DM or that the activity of AIM2 may be regulated by posttranscriptional modifications. Therefore, further investigation into the potential regulatory mechanisms of AIM2 in DM-related inflammatory responses is warranted, as well as whether it influences the expression of CASP1 and IL-1*β* through other pathways.

Genetic and environmental factors play a crucial role in the pathogenesis of DM. This study investigates the effects of temperature, UV exposure, and seasonal variations on serum dsDNA levels in diabetic patients, aiming to further elucidate the potential associations between environmental factors and DM. Previous studies have indicated that as outdoor temperatures rise, the incidence of DM and the prevalence of glucose intolerance increase [27]. Additionally, skin exposure to UV light can facilitate the synthesis of vitamin D, promoting insulin synthesis and secretion, thereby reducing the risk of DM [28]. However, UV radiation may also cause DNA oxidative damage and ssDNA breaks [29]. In terms of seasons, a population-based cohort study in Europe suggested that seasonal changes might influence insulin resistance, leading to elevated blood glucose levels, with higher FPG observed in winter compared to summer [30]. Nevertheless, our findings indicate no significant relationship between serum dsDNA levels in diabetic patients under different temperature, UV exposure, and seasonal conditions. Environmental factors may influence the onset of DM through various mechanisms, but their relationship with serum dsDNA levels requires further investigation.

This study has certain limitations. Our data were solely derived from clinical samples of a hospital in East China. The analysis of environmental factors, such as temperature, season, and UV index, reflects conditions specific to that urban area, and these factors may exhibit significant variations in other regions. Therefore, future research should be conducted across broader regions and more diverse populations to further validate the generalizability of our findings. Our study confirms a correlation between DM and dsDNA, and the findings provide a new perspective for research into the pathogenesis of DM.

## 5. Conclusion

Through experimental analysis, we explored the correlation between DM and dsDNA using peripheral blood serum dsDNA as the focal point. Our findings revealed that serum dsDNA levels were elevated in diabetic patients, and both FPG and HbA1c influenced serum dsDNA levels. This study may provide new therapeutic targets for the onset and progression of DM.

## Figures and Tables

**Table 1:** Basic characteristics of genetic testing in diabetic patients and control groups.

**Group**	**Total (** **N** **)**	**Sex (male/female)**	**Average age (years)**
Healthy control	30	21/9	36
Diabetes mellitus	30	22/8	54

**Table 2:** Results of PSM matching between diabetic patients and the control group.

**Characteristics**	**Before PSM**		**p** ** -value**	**After PSM**		**p** ** -value**
	**Patients**	**Healthy controls**		**Patients**	**Healthy controls**	
No. of patients	339	2970		339	904	
Age (mean SD)	54.04 (14.45)	39.29 (14.16)	< 0.001	54.04 (14.45)	51.53 (13.38)	0.004
Sex (%)			0.273			0.263
Male	204 (60.2)	1690 (56.9)		204 (60.2)	577 (63.8)	
Female	135 (39.8)	1280 (43.1)		135 (39.8)	327 (36.2)	
Season (%)			< 0.001			< 0.001
Summer	108 (31.9)	1598 (53.8)		108 (31.9)	667 (73.8)	
Autumn	39 (11.5)	40 (1.3)		39 (11.5)	6 (0.7)	
Winter	192 (56.6)	1332 (44.8)		192 (56.6)	231 (25.6)	
Temperature (%)			< 0.001			< 0.001
High	108 (31.9)	1598 (53.8)		108 (31.9)	667 (73.8)	
Low	231 (68.1)	1372 (46.2)		231 (68.1)	237 (26.2)	
UV value (%)			< 0.001			< 0.001
High	82 (24.2)	1598 (53.8)		82 (24.2)	667 (73.8)	
Low	257 (75.8)	1372 (46.2)		257 (75.8)	237 (26.2)	
dsDNA (mean SD)	1.09 (0.32)	1.00 (0.24)	< 0.001	1.09 (0.32)	0.97 (0.22)	< 0.001

Abbreviations: dsDNA, double-stranded DNA; PSM, propensity score matching; SD, standard deviation.

## Data Availability

The data that support the findings of this study are available from the corresponding author upon reasonable request.
